# Mortality due to non-AIDS-defining cancers among people living with HIV in Spain over 18 years of follow-up

**DOI:** 10.1007/s00432-023-05500-9

**Published:** 2023-11-26

**Authors:** I. Suárez-García, Félix Gutierrez, José A. Pérez-Molina, Santiago Moreno, Teresa Aldamiz, Eulalia Valencia Ortega, Adrián Curran, Sara Gutiérrez González, Víctor Asensi, Concha Amador Prous, Inma Jarrin, Marta Rava, CoRIS

**Affiliations:** 1 Grupo de Enfermedades Infecciosas, Servicio de Medicina Interna, Hospital Universitario Infanta Sofía, FIIB HUIS HHEN, Madrid, Spain; 2 CIBER de Enfermedades Infecciosas, Instituto de Salud Carlos III, Madrid, Spain; 3 Department of Medicine, Universidad Europea de Madrid, Madrid, Spain; 4 Hospital General Universitario de Elche and Universidad Miguel Hernández, Alicante, Spain; 5 Hospital Universitario Ramón y Cajal, IRYCIS, Madrid, Spain; 6 Hospital Gregorio Marañón Servicio de Enfermedades Infecciosas/Microbiología Clínica Instituto de Investigación Gregorio Marañón, Madrid, Spain; 7 Hospital Universitario La Paz, Madrid, Spain; 8 Infectious Diseases Department, Vall d’Hebron Research Institute (VHIR), Hospital Universitari Vall d’Hebron, Barcelona, Spain; 9 Servicio de Medicina Interna, Hospital Clínico Universitario de Valladolid, Valladolid, Spain; 10 Infectious Diseases Unit, Hospital Universitario Central de Asturias and Group of Translational Research in Infectious Diseases, Instituto de Investigación Sanitaria del Principado de Asturias (ISPA), Oviedo, Spain; 11 Hospital de La Marina Baixa, Alicante, Spain; 12 Centro Nacional de Epidemiologia, Instituto de Salud Carlos III, Madrid, Spain

**Keywords:** Cancer, Mortality, HIV, Cohort study

## Abstract

**Purpose:**

Our aim was to describe non-AIDS-defining cancer (NADC) mortality among people living with HIV (PLWH), to compare it with that of the general population, and to assess potential risk factors.

**Methods:**

We included antiretroviral-naive PLWH from the multicentre CoRIS cohort (2004–2021). We estimated mortality rates and standardised mortality ratios (SMRs). We used cause-specific Cox models to identify risk factors.

**Results:**

Among 17,978 PLWH, NADC caused 21% of all deaths observed during the follow-up. Mortality rate due to NADC was 1.58 (95%CI 1.36, 1.83) × 1000 person-years and lung and liver were the most frequent cancer-related causes of death. PLWH had 79% excess NADC mortality risk compared to the general population with the highest SMR found for Hodgkin lymphoma, anal and liver cancers. The SMRs decreased with age and were the highest in age groups under 50 years. The most important prognostic factor was low CD4 count, followed by smoking, viral hepatitis and HIV transmission through heterosexual contact or injection drug use.

**Conclusion:**

Non-AIDS cancers are an important cause of death among PLWH. The excess mortality related to certain malignancies and the association with immunodeficiency, smoking, and coinfections highlights the need for early detection and treatment of cancer in this population.

**Supplementary Information:**

The online version contains supplementary material available at 10.1007/s00432-023-05500-9.

## Introduction

Cancers account for a considerable proportion of morbidity and mortality among people living with HIV (PLWH) (Engels et al. 2017). With the widespread use of effective combination antiretroviral therapy (ART), there was a shift in the predominant types of cancers that affect this population, from AIDS-defining cancers, which include those related to advanced immunodeficiency that characterise AIDS (Kaposi’s sarcoma, non-Hodgkin lymphoma, and invasive cervical cancer) to non-AIDS–defining cancers (NADC) (Shiels and Engels 2017).

During the last decades, mortality rates in PLWH have decreased for many causes of death, including AIDS cancers, while NADC mortality has remained constant (Weber et al. 2013; Smith et al. 2014) becoming one of the leading causes of death in PLWH (Croxford et al. 2017; Yuan et al. 2022). Meanwhile, ART has improved its effectiveness, cancer prevention and control have increased due to the human papillomavirus (HPV) vaccine and hepatitis C virus (HCV) treatment as well as screening and prevention campaigns, and, finally, treatment strategies for cancer have evolved. In this setting, quantifying and monitoring cancer mortality trends in PLWH are essential to understand its evolution and the underlying risk factors with the final aim of highlighting targeted priorities in care and prevention. In Spain, NADC mortality was three times higher among PLWH than the general population during the years 1999–2006 (Aldaz et al. 2011), but data on recent mortality trends over time are lacking or only limited to the regional level (López et al. 2016).

Factors potentially contributing to increasing incidence of NADC among PLWH include traditional cancer risk factors, such as aging and smoking, among others, as well as HIV-dependent risk factors (including length of HIV infection and immunosuppression, (Clifford et al. 2005) co-infections with HPV, hepatitis B virus (HBV) or HCV, (Picard et al. 2016; Hu et al. 2019) immune system activation and chronic inflammation (Tenorio et al. 2014)). However, there is little evidence on specific risk factors for NADC mortality among PLWH, and the few studies that have investigated them (Bonnet et al. 2004; d’Arminio et al. 2008; Leierer et al. 2021) have not considered multiple risk factors including HIV-related and -unrelated. The only study that investigated risk factors for cancer mortality among PLWH that considered both traditional and HIV-related risk factors was limited to the years 1998–2001 (d’Arminio et al. 2008).

The aims of this study were to describe NADC mortality and its temporal trends among PLWH, to compare them with those in the general population, and to assess potential risk factors during the years 2004 to 2021.

## Materials and methods

### Study population

CoRIS is an open, prospective, multicentre cohort of confirmed HIV-positive individuals. Participants are ART-naive at study entry, recruited in 47 clinical centres from 14 of the 17 autonomous regions in Spain from 2004 onwards. In brief, CoRIS collects a dataset including baseline and follow-up sociodemographic, immunological and clinical data. Data are highly standardized and submitted to periodic quality control procedures. Participants are followed up periodically following routine clinical practice (Sobrino-Vegas et al. 2011).

For this study, we included antiretroviral-naïve individuals aged ≥ 20 years at enrolment and with at least one day of follow-up, recruited from January 1st, 2004 to November 30th, 2021, which was considered as administrative censoring date.

All patients agree to participate in CoRIS by signing an informed consent form. The CoRIS cohort was approved by the Clinical Research Ethics Committee of the Gregorio Marañón General University Hospital. This study was approved by the Carlos III Institute Research Ethics Committee (PI 05_2019) and was performed in accordance with the Declaration of Helsinki.

The data that support the findings of this study are available on request from the corresponding author. The data are not publicly available due to privacy or ethical restrictions.

### Outcomes

The main outcome was death due to NADC. We also considered the following endpoints: viral and nonviral NADC mortality, mortality due to lung cancer and mortality due to liver cancer. Deaths were ascertained through cohort reporting, classified using revised CoDe, which is a simplified version of CoDe coding system that has been proposed by the Antiretroviral Therapy Cohort Collaboration (Zwahlen et al. 2009), which has been previously applied to CoRIS (Hernando et al. 2012).

According to different pathogenesis and risk factors, we further categorized NADC deaths as viral, nonviral, and other (Hernández-Ramírez et al. 2017). Viral NADC deaths included those due to cancers of the anus and penis which are related to HPV; liver cancer, which is associated with HBV and HCV; Hodgkin’s lymphoma, which is related to Epstein–Barr virus; and Merkel cell carcinoma, which is associated with Merkel cell polyomavirus. Cancers of vagina/vulva and oral cavity/pharynx, which are caused by HPV, were categorized together with other nonviral cancers (such as gynaecological or head and neck cancers) in the database. Since they could not be classified as viral or nonviral NADC, gynaecological, head and neck as well as cancers of unknown primary site were classified as ‘other’.

### Statistical analyses

We estimated 5-years age group- and sex-specific annual mortality rates as the number of deaths due to NADC by 1000 person-years of follow-up with 95% confidence intervals (CIs) based on the normal approximation of the logarithm of the rates. Follow-up of individuals began on the date of enrolment to the date of death, last visit or administrative censoring date, whichever came first.

To compare the cohort mortality to that of the general population, we estimated age-and sex-specific annual Standardized Mortality Ratios (SMR), defined as the ratio of observed to expected deaths according to mortality among the general population. Expected deaths were obtained by applying calendar year, sex- and age-specific (in 5-years bands) mortality rates for the general population to the person-years of follow-up of the CoRIS cohort. We obtained the annual cause-specific mortality rates for the general population from the National Statistics Institute (www.ine.es) during 2004–2021. We performed a test for trend by including age or period as a continuous variable and a test for interaction by including sex as an interaction term, both in a Poisson regression model. We finally obtained age-, sex- and period-(2004–2006, 2007–2010, 2011–2013, 2014–2017, 2018–2021) specific rates and SMR. We chose the year 2014 as one of the cut-off points because it was in that year when the Spanish antiretroviral treatment guidelines changed to recommend initiation of ART regardless of the CD4 cell count.

We estimated mortality rate ratios (MRR) for mortality due to all NADC, viral, nonviral NADC, lung and liver cancers, using cause-specific Cox proportional hazard models (to account for competing risk events), with age as timescale. We estimated robust standard errors to account for clustering of participants within centres. In each model we included a set of baseline and time-updated variables as potential prognostic factors. Baseline variables were sex at birth (male, female), transmission route (males having sex with other males [MSM], injecting drug use [IDU], heterosexual contact, other/unknown), educational level (no/primary education, secondary education, university, other/unknown), region of origin (Europe, Sub-Saharan Africa, Latin America, other/unknown) and AIDS diagnosis at enrolment. Time-updated variables were CD4 T-cell count (< 200, 201–349, 350–499, ≥ 500 cells/µL, unknown), presence of HCV antibodies (no, yes or unknown) or hepatitis B surface antigen (no, yes or unknown), CD4/CD8 ratio (≤ 0.4, > 0.4, unknown), viral load (< 50, 51–10,000, 10,000–100,000, ≥ 100,000 copies/mL, unknown) and smoking status (never, ex-, current smoker, unknown).

Statistical analyses were performed using R version 4.2 (R Core Team 2020).

## Results

Among the 18,573 participants included in CoRIS by November 2021, 17,978 met inclusion criteria: 85.5% of them were men, the majority was from Spain (55.2%) and the most frequent mode of HIV acquisition was MSM (62.6%) (Table 1). At enrolment, median age was 35 years, 33.4% subjects had CD4 T-cell count ≥ 500 cells/µL, and 12.4% had an AIDS diagnosis. Among subjects with known smoking status, 5,357 (44.0%) were active smokers.

**Table 1:** Baseline characteristics of participants included in the study and of those who died due to a non-AIDS defining cancer, CoRIS cohort, 2004–2021

	All participants (*N* = 17,978)	Deaths due to NADC (*N* = 176)
*Sex*		
Female	2604 (14.5%)	29 (16.5%)
Male	15,374 (85.5%)	147 (83.5%)
*Age at enrolment, years*		
Median (Q1, Q3)	35.4 (29.1, 43.1)	47.8 (41.9, 54.7)
< 40	11,199 (62.3%)	33 (18.8%)
40–49	4463 (24.8%)	68 (38.6%)
≥ 50	2316 (12.9%)	75 (42.6%)
*Transmission route*		
Men having sex with men	11,248 (62.6%)	52 (29.5%)
Injecting drug user	1206 (6.7%)	43 (24.4%)
Heterosexual	4843 (26.9%)	72 (40.9%)
Other/Unknown	681 (3.8%)	9 (5.1%)
*Educational level*		
None or primary education	2277 (12.7%)	33 (18.8%)
Secondary education/University	12,485 (69.4%)	111 (63.1%)
Other/Unknown	3216 (17.9%)	32 (18.2%)
*Region of origin*		
Europe	12,752 (70.9%)	158 (89.8%)
Sub-Saharan Africa	784 (4.4%)	5 (2.8%)
Latin America	4053 (22.5%)	10 (5.7%)
Other/Unknown	389 (2.2%)	3 (1.7%)
*Smoking status*		
Never smoker	5611 (31.2%)	24 (13.6%)
Smoker	5357 (29.8%)	74 (42.0%)
Ex-Smoker	1207 (6.7%)	17 (9.7%)
Unknown	5803 (32.3%)	61 (34.7%)
*CD4, cell/µL*		
Median (Q1, Q3)	389.0 (210.0, 580.5)	245.0 (102.0, 419.0)
< 200	4126 (23.0%)	69 (39.2%)
200–349	3643 (20.3%)	40 (22.7%)
350–499	3804 (21.2%)	32 (18.2%)
≥ 500	6002 (33.4%)	30 (17.0%)
Unknown	403 (2.2%)	5 (2.8%)
*Viral load, copies/ml*		
< 10,000	4226 (23.5%)	36 (20.5%)
10, 000–100, 000	7056 (39.2%)	68 (38.6%)
≥ 100, 000	6264 (34.8%)	66 (37.5%)
Unknown	432 (2.4%)	6 (3.4%)
*CD4/CD8*		
≤ 0.4	6618 (36.8%)	82 (46.6%)
> 0.4	6872 (38.2%)	44 (25.0%)
Unknown	4488 (25.0%)	50 (28.4%)
AIDS diagnosis	2231 (12.4%)	51 (29.0%)
*Hepatitis C virus antibodies*		
No	14,768 (82.1%)	105 (59.7%)
Yes	1694 (9.4%)	60 (34.1%)
Unknown	1516 (8.4%)	11 (6.2%)
*Hepatitis B surface antigen*		
No	13,642 (75.9%)	143 (81.2%)
Yes	595 (3.3%)	10 (5.7%)
Unknown	3741 (20.8%)	23 (13.1%)

*NADC* non-AIDS defining cancers

### Mortality rates due to NADC

We observed that 176 of the 851 (20.7%) deaths registered during the study period were due to NADC: of these, 31 were due to a viral (17.6%), 125 (71.0%) were due to a nonviral cancer and 20 (11.4%) were due to other cancers. Mortality rates and SMR due to all and specific NADC are shown in Table 2.

**Table 2:** NADC mortality rate and standardized mortality ratios by cancer type: All NADC, viral NADC, nonviral NADC and other (person-years = 111,325)

Type	Observed (*N*)	Rate × 1000 person-years 95%CI	Expected (*N*)	SMR 95%CI
All NADC	176	1.58 (1.36, 1.83)	98.36	1.79 (1.54, 2.07)
Viral NADC	31 (19.9%)	0.28 (0.20, 0.40)	6.43	4.82 (3.39, 6.85)
Liver cancer	22 (12.5%)	0.20 (0.13, 0.30)	5.74	3.83 (2.52, 5.82)
Hodgkin lymphoma	7 (4%)	0.06 (0.03, 0.13)	0.37	19.10 (9.10, 40.05)
Anal cancer	2 (1.1%)	0.02 (0.00, 0.07)	0.17	12.05 (3.01, 48.20)
Nonviral NADC	125 (80.1%)	1.12 (0.94, 1.34)	81.28	1.54 (1.29, 1.83)
Lung cancer	64 (36.4%)	0.57 (0.45, 0.73)	27.95	2.29 (1.79, 2.93)
Colorectal cancer	12 (6.8%)	0.11 (0.06, 0.19)	11.29	1.06 (0.60, 1.87)
Stomach cancer	8 (4.5%)	0.07 (0.04, 0.14)	5.14	1.56 (0.78, 3.11)
Oesophagus cancer	7 (4%)	0.06 (0.03, 0.13)	2.98	2.35 (1.12, 4.93)
Pancreas cancer	6 (3.4%)	0.05 (0.02, 0.12)	6.22	0.96 (0.43, 2.15)
Kidney cancer	5 (2.8%)	0.04 (0.02, 0.11)	2.36	2.12 (0.88, 5.09)
Other malignancy type*	5 (2.8%)	0.04 (0.02, 0.11)	4.88	1.02 (0.43, 2.46)
Leukaemia	4 (2.3%)	0.04 (0.01, 0.10)	2.54	1.57 (0.59, 4.20)
Bladder cancer	3 (1.7%)	0.03 (0.01, 0.08)	3.06	0.98 (0.32, 3.04)
Prostate cancer	3 (1.7%)	0.03 (0.01, 0.08)	2.96	1.01 (0.33, 3.14)
Breast cancer	2 (1.1%)	0.02 (0.00, 0.07)	2.85	0.70 (0.18, 2.81)
Malignant melanoma	2 (1.1%)	0.02 (0.00, 0.07)	1.34	1.49 (0.37, 5.95)
Multiple myeloma	2 (1.1%)	0.02 (0.00, 0.07)	1.07	1.86 (0.47, 7.44)
Brain cancer	1 (0.6%)	0.01 (0.00, 0.06)	4.70	0.21 (0.03, 1.51)
Connective tissue cancer	1 (0.6%)	0.01 (0.00, 0.06)	0.88	1.14 (0.16, 8.10)
Other cancers				
Unknown	15 (8.5%)	0.13 (0.08, 0.22)	3.52	4.27 (2.57, 7.08)
Head and neck (incl. face) cancers	5 (2.8%)	0.04 (0.02, 0.11)	6.04	0.83 (0.34, 1.99)

*NADC* non-AIDS defining cancers, *SMR* standardized mortality ratios

*Other malignancy type: Carcinoma of hepatobiliary origin (*n* = 1), Lymphoma (*n* = 1), Neuroendocrine tumours (*n* = 1) and Sarcomas (*n* = 2)

NADC mortality rate was 1.58 (95% 1.36, 1.83): the highest mortality rates were found for lung and liver cancer.

Mortality rates due to both viral and nonviral cancers were similar by sex (P-value for interaction, [Pint] = 0.061) and increased with age (*P*-value for trend, [Ptrend] < 0.0001). The mortality rates for nonviral cancers were stable over the follow-up period, whereas those for viral cancers showed a decrease from the years 2004–2006 to the years after 2007. We also observed a slight increase during 2018–2021, which was mainly due to an increase in liver cancer mortality rates which varied from 0.15 × 1,000 person-years (95%CI 0.06–0.40) in 2014–2017 to 0.34 × 1,000 person-years (95%CI 0.20–0.59) during 2018–2021 (see online resource Fig. 1).

**Fig. 1:**
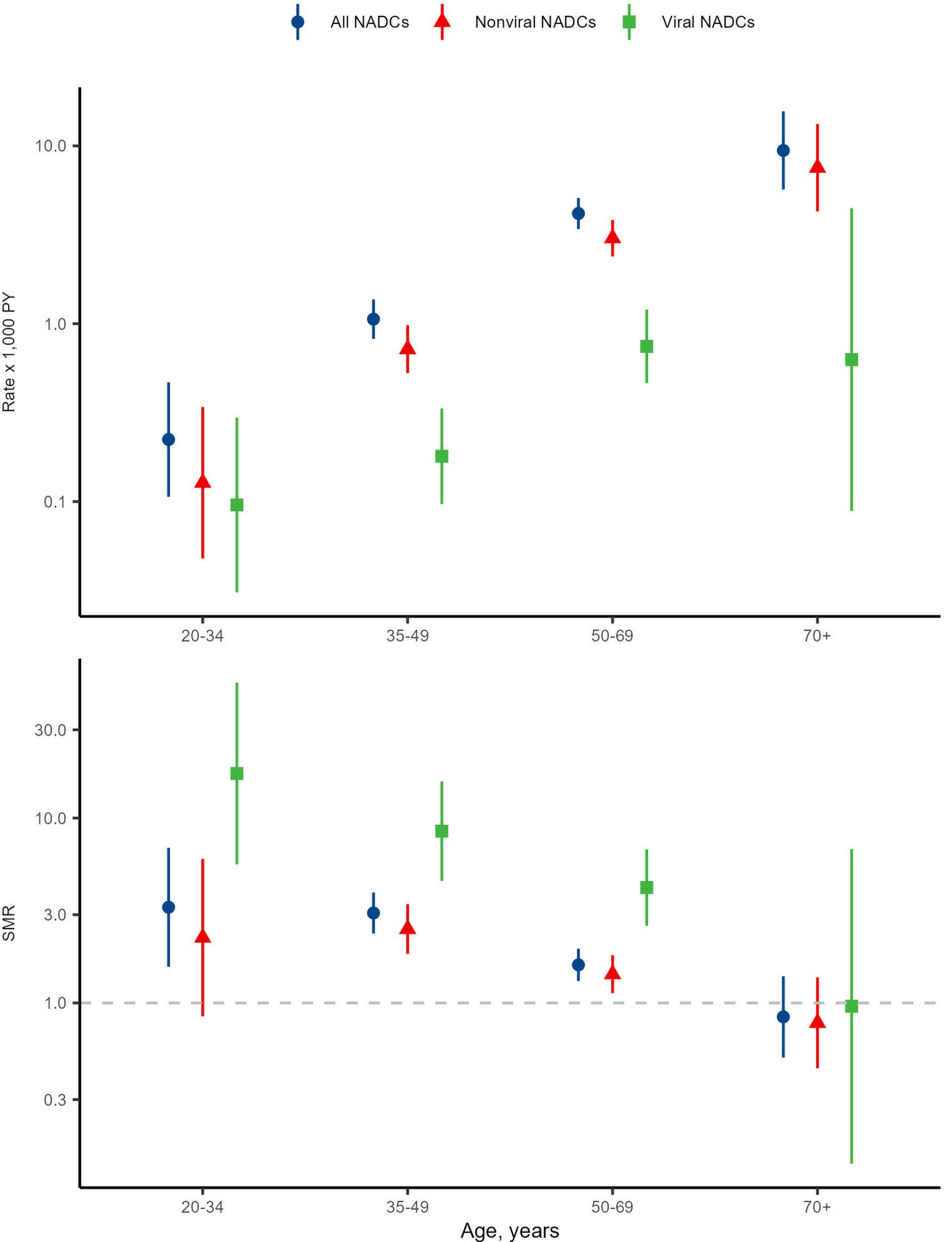
Non-AIDS defining cancer mortality rates and standardized mortality ratios by age for all NADC, nonviral and viral NADC. *NADC* non-AIDS defining cancers, *PY* person-years, *SMR* standardized mortality ratios

NADC mortality was higher among PLWH than that of the general population, matched by age, sex and period (SMR: 1.79 (95%CI 1.54, 2.07)), especially for viral NADC and for most specific cancers (Table 2). SMRs were highest for Hodgkin lymphoma, anal and liver cancer. The risk of death due to oesophageal, kidney or lung cancer among PLWH was at least twice that of the general population. Whenever mortality was lower in PLWH than that of the general population, the estimates were imprecise.

SMRs for viral cancers were higher in females (17.35 (95%CI 7.79, 38.61)) than in males (4.11 (95%CI 2.77, 6.08), P_int_ = 0.007). For nonviral cancers, there was some evidence that the SMRs were also higher in females than males (2.23 (95%CI 1.45, 3.42) vs 1.45 (95%CI 1.19, 1.75), P_int_ = 0.086) (See online resource Table 1).

The SMRs decreased with age: NADC mortality in PLWH was three times higher than that of the general population in age groups under 50 years and gradually decreased to a similar rate than that of the general population in age groups over 70 years (*P*_trend_ < 0.001). This decrease was particularly evident for viral cancers (*P*_trend_ = 0.054). (Fig. 1). Like the mortality rates, SMRs for viral cancers were the highest during the years 2004–2006 and decreased thereafter while SMRs for nonviral and all NADC showed minor variation over time (*P*_trend_ = 0.077 and 0.083, respectively) (Online resource Fig. 1).

### Participants’ characteristics in association with NADC mortality

Table 3 shows the multivariable analysis of participants’ characteristics associated with mortality due to all, viral and nonviral NADC, and lung and liver cancers. Subjects who acquired HIV through heterosexual contact or use of injected drugs had a higher mortality risk due to NADC than men who acquired HIV through sex with men. The factor that was most strongly associated with mortality in all categories analysed (all NADC, and viral, nonviral and lung cancers), was the time-updated CD4 T-cell count. Compared to subjects with CD4 counts ≥ 500/µL, the mortality risk steeply increased with more profound immunodeficiency. Active smoking was strongly associated with mortality due to all, nonviral, and lung cancers, and hepatitis B or C infection were associated with NADC mortality in all categories. For all categories analysed, there was some evidence that subjects from non-European origin had a lower risk of mortality. After adjusting for other risk factors, mortality in all categories analysed was similar for sex, viral load, CD4/CD8 ratio, or AIDS diagnosis at enrolment. Lung cancer mortality risk increased with active or past smoking, heterosexual contact as transmission route, and CD4 counts < 500/µL; liver cancer mortality was associated with injected drugs or heterosexual contact as transmission route.

**Table 3:** Association between participants’ characteristics and non-AIDS defining cancer mortality

	Adjusted MRR (95%CI)				
	All NADC	Nonviral NADC	Lung	Viral NADC	Liver
Males vs females	1.17 (0.76, 1.80)	1.11 (0.77, 1.62)	1.66 (0.87, 3.16)	1.08 (0.38, 3.03)	2.17 (0.83, 5.70)
Transmission route, ref. MSM	1				
Injecting drug user	1.86 (1.02, 3.39)	1.92 (0.79, 4.66)	2.64 (0.86, 8.07)	2.28 (0.59, 8.88)	27.80 (2.00, 386.71)
Heterosexual	1.51 (1.08, 2.13)	1.40 (0.91, 2.16)	2.63 (1.16, 5.97)	1.69 (0.60, 4.81)	8.86 (2.22, 35.40)
Other/Unknown	1.20 (0.60, 2.41)	1.46 (0.67, 3.18)	3.33 (0.88, 12.61)	0.00 (0.00, 0.00)	0.00 (0.00, 0.00)
Educational level, ref. None/primary	1				
Secondary/university	1.49 (0.85, 2.60)	1.27 (0.67, 2.42)	0.68 (0.39, 1.17)	4.72 (1.45, 15.38)	3.87 (0.79, 19.02)
Other/Unknown	1.32 (0.88, 1.98)	1.20 (0.77, 1.86)	0.55 (0.24, 1.26)	1.10 (0.25, 4.80)	0.17 (0.02, 1.75)
Region of origin, ref. Europe	1				
Sub-Saharan Africa	0.78 (0.31, 1.94)	Extra-Europe*:	Extra-Europe*:	Extra-Europe*:	Extra-Europe*:
Latin America	0.51 (0.23, 1.11)	0.68 (0.35, 1.30)	0.42 (0.11, 1.60)	0.81 (0.27, 2.46)	0.19 (0.02, 1.76)
Other/Unknown	1.14 (0.44, 2.98)				
Smoking status^#^, ref. Never smoker	1	1		1	1
Smoker	2.23 (1.05, 4.70)	3.51 (1.12, 10.99)	18.20 (3.28, 101.14)	1.09 (0.33, 3.64)	0.67 (0.14, 3.23)
Ex-Smoker	1.68 (0.81, 3.46)	2.62 (0.88, 7.87)	7.06 (1.11, 44.83)	0.61 (0.13, 2.99)	0.32 (0.05, 2.18)
Unknown	1.05 (0.43, 2.60)	1.45 (0.42, 4.92)	9.53 (1.80, 50.54)	0.52 (0.19, 1.43)	1.35 (0.38, 4.81)
CD4^#^, cell/µL, ref. ≥ 500					
≤ 200	9.76 (5.35, 17.80)	8.74 (4.10, 18.63)	2.39 (1.06, 5.38)	21.65 (4.67, 100.31)	1.91 (0.42, 8.65)
200–349	4.17 (2.31, 7.54)	4.68 (2.28, 9.59)	1.64 (0.88, 3.06)	3.16 (0.71, 14.12)	1.96 (0.72, 5.29)
350–499	2.61 (1.62, 4.22)	2.42 (1.37, 4.30)	1.59 (1.16, 2.19)	5.31 (2.23, 12.64)	1.58 (1.02, 2.45)
Unknown	4.70 (1.32, 16.70)	5.57 (1.48, 20.93)	1.44 (0.29, 7.21)	0.00 (0.00, 0.00)	3.67 (0.79, 17.04)
Viral load^#^, copies/ml, ref < 50					
51–10,000	1.04 (0.63, 1.72)	0.50 (0.26, 0.99)	1.07 (0.82, 1.40)	1.89 (0.78, 4.56)	0.89 (0.51, 1.58)
≥ 10,000	0.76 (0.43, 1.34)	0.88 (0.54, 1.43)	1.30 (0.85, 1.99)	0.55 (0.16, 1.87)	1.61 (0.79, 3.28)
Unknown	0.44 (0.12, 1.65)	0.52 (0.15, 1.81)	2.55 (0.57, 11.42)	0.00 (0.00, 0.00)	3.45 (0.87, 13.68)
CD4/CD8^#^, ref > 0,4					
≤ 0,4	0.73 (0.43, 1.23)	0.67 (0.36, 1.26)	1.46 (0.80, 2.64)	0.52 (0.16, 1.77)	0.71 (0.21, 2.44)
Unknown	0.66 (0.41, 1.06)	0.63 (0.35, 1.13)	1.58 (0.57, 4.34)	0.53 (0.22, 1.29)	0.23 (0.04, 1.37)
AIDS diagnosis at enrolment	1.12 (0.85, 1.47)	1.14 (0.83, 1.55)	0.94 (0.53, 1.68)	1.11 (0.56, 2.21)	1.34 (0.68, 2.64)
Hepatitis C virus antibodies^#^, ref No					
Yes	1.59 (0.98, 2.57)	1.17 (0.59, 2.33)	1.46 (0.71, 3.02)	4.04 (1.69, 9.62)	2.49 (0.25, 24.47)
Unknown	2.14 (1.31, 3.49)	2.63 (1.33, 5.23)	3.05 (1.35, 6.89)	0.96 (0.27, 3.40)	0.78 (0.11, 5.77)
Hepatitis B surface antigen^#^, ref No					
Yes	2.03 (1.22, 3.38)	1.62 (0.80, 3.29)	1.62 (0.61, 4.32)	3.59 (0.93, 13.85)	3.63 (0.88, 15.08)
Unknown	0.68 (0.42, 1.10)	0.59 (0.34, 1.05)	0.56 (0.27, 1.14)	1.27 (0.56, 2.87)	1.86 (1.07, 3.24)

*CI* confidence interval, *MRR* Mortality rate ratio, *MSM* males having sex with other males

Adjusted MRR (CI 95%) were estimated with extended cause-specific Cox regression models with age as timescale

*For nonviral, viral, lung and liver cancer region of origin was grouped as Europe vs extra-Europe

^#^modelled as time-updated variables

## Discussion

In this large Spanish cohort of PLWH followed up between 2004 and 2021, we observed that NADC accounted for 21% of all deaths and that NADC mortality rate was 1.58 per 1,000 person-years. We observed that PLWH had a 79% higher risk of NADC mortality than the general population, especially among women and at younger ages. Sustained immunodeficiency, smoking and viral hepatitis were independently associated with an increased risk of NADC mortality. These findings underline that PLWH have an increased risk of death due to NADC than people from the general population of the same sex, age and period and that NADC mortality risk is related to a combination of traditional life-style factors, such as smoking and coinfection, and HIV-related risk factors, such as immunodeficiency. This is, to our knowledge, the first study assessing risk factors for NADC mortality that considers both traditional and HIV-related factors in recent years.

Although mortality in PLWH has been decreasing during the last decade in most European countries including Spain (Gueler et al. 2017; Fontela et al. 2020), we observed that NADC mortality rates remained constant over time. Similar results were observed in a multi-cohort study including PLWH from Europe, USA, and Australia during 1999–2011 (Smith et al. 2014) and more recently in the Navarra region in Spain (Fontela et al. 2020). Earlier European studies have shown a stable incidence of NADC among PLWH (Franceschi et al. 2010; García-Abellán et al. 2019; Poizot-Martin et al. 2021) and the fact that NADC mortality rates did not decrease in PLWH, while they did in the general population (Sociedad Española de Oncología Médica 2023), suggests that cancer-reducing interventions implemented so far might not be adequately reaching PLWH.

NADC mortality was 80% higher than that of the general Spanish population: this is similar to that reported in other studies (Croxford et al. 2017; Hessol et al. 2018). SMRs were the highest for cancers related to oncogenic viruses, but also for smoking-related cancers including lung, oesophagus and kidney, as previously observed (Yuan et al. 2022). Results from this same cohort during the years 2004–2014 have shown that the NADC excess mortality with respect to the general population was mainly due to increasing age, low CD4 count and HCV coinfection (Alejos et al. 2016). The same factors that are associated with cancer incidence may explain this increased mortality risk, such as smoking, alcohol use, chronic viral hepatitis, and other pro-oncogenic viruses such as HPV (Parka et al. 2016; Leierer et al. 2021). Also, PLWH have shown poorer outcomes compared with HIV-negative oncological patients (Suneja et al. 2013), which could be related to more advanced cancer stage at diagnosis (Shiels et al. 2015) or disparities in cancer treatment (Suneja et al. 2016) and screening (Corrigan et al. 2019). Most of these studies have been conducted in the United States, and whether the same drivers apply also in Europe is largely unknown.

We observed higher SMRs for women than for men, mainly for viral cancer mortality, in contrast with a study in the United Kingdom (Croxford et al. 2017) that reported slightly higher SMRs in males, but only during the first year of diagnosis.

Previous studies observed higher SMR for all-cause mortality in women than in men (Lewden et al. 2012). This result may reflect differences in the distribution of factors associated with NADM mortality (such as education, socioeconomic status and access to health care, prevalence of smoking, alcohol consumption, use of injected drugs or coinfections) between women living with HIV and those of the general population. In Spain, women living with HIV have been infected through use of injected drugs more frequently than men, and therefore have a higher prevalence of hepatitis C coinfection, as we have previously seen in our cohort (Muñoz Hornero et al. 2021). The excess mortality due to viral cancers in women is likely due to hepatocellular carcinoma and is probably due to the higher prevalence of chronic hepatitis B and C infection observed in women living with HIV with respect to those HIV-seronegative as observed in Collins et al. (Collins et al. 2021).

There is a lack of research investigating specific risk factors for NADC mortality in women with HIV.

The greatest difference in mortality risk between PLWH and the general population occurred before 50 years of age. Previous studies have shown younger ages at diagnosis in PLWH for certain cancers, such as lung and anal (Shiels et al. 2017), which could be related to the earlier and/or greater exposure to risk factors such as cigarette smoking and HPV (Parka et al. 2016). Also, PLWH with HCV or HBV coinfection experience faster progression to cirrhosis (Osborn et al. 2007; de Lédinghen et al. 2008) which is the main risk factor for hepatocellular carcinomas.

Results from the multivariable analysis showed that, compared to MSM, mortality risk was higher in subjects who acquired HIV through heterosexual contact and especially through IDU. This could be related to earlier diagnosis due to active HIV testing and a good adherence to HIV management that are more frequent in MSM (Rava et al. 2021; Izquierdo Miguel et al. 2023). The higher mortality due to NADC in IDU has been previously described (Trickey et al. 2016).

There was some evidence of lower NADC mortality among subjects of non-European origin. A previous meta-analysis showed lower mortality in migrants with respect to general population for all-cause mortality as well as for cancer mortality (Aldridge et al. 2018). These results can be explained with the healthy migrant hypothesis (people that decide to migrate are usually young and healthy) and the salmon bias (migrants frequently return to their country of origin when they are in poor health or before death) (Borhade and Dey 2018).

Two potentially modifiable factors were most strongly associated with mortality: low CD4 count and smoking. CD4 count was the most important prognostic factor for mortality due to all, viral, and nonviral NADC, including lung cancer. This has been observed in previous studies (d’Arminio et al. 2008; Leierer et al. 2021) and underlines the need for prompt HIV diagnosis and early treatment initiation, as well as good treatment adherence. Besides, these associations support the implementation of tailored cancer surveillance approach in PLWH with higher risk of NADC incidence and mortality. Regarding smoking, 44% of our patients were active smokers, which is a higher proportion than that of the Spanish general population, which ranged between 29.6% and 34.0% during the years 2005–2019 (Observatorio Español de las Drogas y las Adicciones 2021). Given that active smoking was associated with a two-fold risk of NADC mortality and more than ten-fold risk of lung cancer mortality, and that more than a third of the NADC deaths were due to lung cancer, this stresses the need to reinforce the interventions for smoking cessation among PLWH.

After adjusting for other risk factors, other markers of advanced HIV infection as HIV viral load, AIDS diagnosis at enrolment, and CD4/CD8 ratio were not associated with mortality. A study in the D:A:D cohort (Monforte et al. 2008a) also found that HIV viral load was not significantly associated with mortality after adjusting for CD4 count.

Hepatitis B and C coinfections were associated with an increased risk of death due to NADC, and specifically hepatitis C was associated with increased mortality due to viral cancers. However, although the risk estimates for liver cancer mortality were high for patients with hepatitis B or C, the estimates were uncertain probably due to the low number of cases. Our results are also limited by the lack of information on direct acting antivirals for the treatment of hepatitis C, which have been shown to decrease the incidence of liver cancer (Morgan et al. 2013).

Our study has several limitations. We were not able to ascertain the cause of death in 11.4% of the subjects. Also, we could not include in the viral cancer group several cancers of known viral origin such as vagina/vulva and oral cavity/pharynx, which are caused by HPV. Another limitation was the lack of information on HPV infection as well as the high proportion of missing data for important cancer-associated factors such as BMI, alcohol consumption, smoking status and HCV/HBV coinfections.

In conclusion, NADC mortality accounted for a large proportion of deaths among PLWH and was associated mainly to immunodeficiency, smoking, and coinfection with hepatitis B and C, which are all preventable risk factors. Besides, NADC mortality was higher in PLWH than in the general population, especially for viral cancers and among women and at younger ages. These findings highlight the importance of prompt HIV diagnosis and linkage to care, along with tailored strategies to enhance early detection of cancer as well as the need of prevention and modification of traditional cancer risk factors among PLWH, with a special attention to women and younger people.

## Supplementary Information

Below is the link to the electronic supplementary material.

Supplementary file1 (PDF 257 KB)

Supplementary file2 (PDF 155 KB)

## Data Availability

The datasets generated and/or analysed during the current study are available from the corresponding author on reasonable request.

## Abbreviations

ART: Antiretroviral therapy
HBV: Hepatitis B virus
HCV: Hepatitis C virus
HPV: Human papillomavirus
IDU: Injecting drug use
MRR: Mortality rate ratio
MSM: Men having sex with men
NADC: Non-AIDS-defining cancers
PLWH: People living with HIV
SMR: Standardized mortality ratio
