# Supplementary material for: Mortality due to non-AIDS-defining cancers among people living with HIV in Spain over 18 years of follow-up
Source: J Cancer Res Clin Oncol. 2023 Nov 26;149(20):18161–71. doi: 10.1007/s00432-023-05500-9 (PMC10725373; doi:10.1007/s00432-023-05500-9)
Supplement: Supplementary file 1 — Supplementary file1 (PDF 257 KB) [file 432_2023_5500_MOESM1_ESM.pdf]

Mortality due to non-AIDS-defining cancers among people living with HIV in Spain over 18 years of follow-up

Journal of Cancer Research and Clinical Oncology

Corresponding author:

Marta Rava

Centro Nacional de Epidemiología
Instituto de Salud Carlos III
Avenida Monforte de Lemos 5, 28029 Madrid
Teléfono: +34 918 222 869
e-mail: mrava@isciii.es
orcid id: 0000-0003-2260-9370

Supplementary information

Table 1*: Rates and standardized mortality rates by sex*

	Males, person-years= 92,955				Females, person-years= 18,370			
	**N**	**Rate x1000 person-years (95%CI)**	**Expected (N)**	**SMR 95%CI**	**Observed (N)**	**Rate x1000 person-years 95%CI**	**Expected (N)**	**SMR 95%CI**
**All**	147	1.58 (1.35, 1.86)	86.86	1.69 (1.44, 1.99)	29	1.58 (1.10, 2.27)	11.50	2.52 (1.75, 3.63)
**Nonviral**	104	1.12 (0.92, 1.36)	71.85	1.45 (1.19, 1.75)	21	1.14 (0. 75, 1.75)	9.42	2.23 (1.45, 3.42)
**Viral**	25	0.27 (0.18, 0.40)	6.09	4.11 (2.77, 6.08)	6	0.33 (0.15, 0.73)	0.35	17.35 (7.79, 38.61)

***Fig. 1**: NADC mortality rates and SMR by period for all NADC, nonviral and viral NADC*

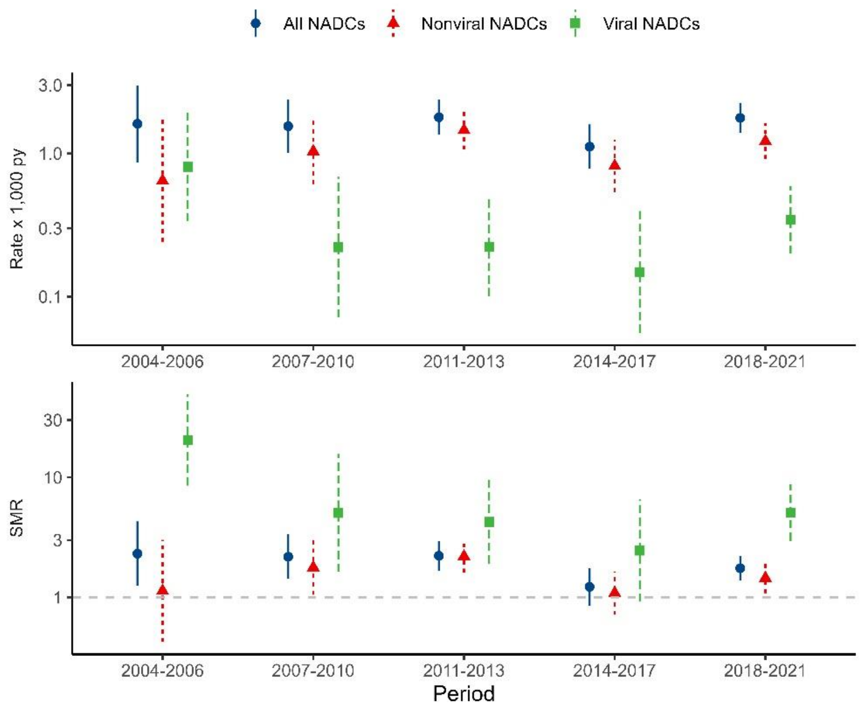

NADC: non-AIDS defining cancers; PY: person-years; SMR: standardized mortality ratios
